# Predicting the Fate of Biodiversity Using Species’ Distribution Models: Enhancing Model Comparability and Repeatability

**DOI:** 10.1371/journal.pone.0044402

**Published:** 2012-09-11

**Authors:** Genoveva Rodríguez-Castañeda, Anouschka R. Hof, Roland Jansson, Larisa E. Harding

**Affiliations:** 1 Ecology and Evolution, Stony Brook University, Stony Brook, New York, United States of America; 2 Landscape Ecology Group, Department of Ecology and Environmental Science, Umeå University, Umeå, Sweden; 3 Department of Wildlife, Fish and Environmental Studies, Swedish University of Agricultural Sciences (SLU), Umeå, Sweden; University of Utah, United States of America

## Abstract

Species distribution modeling (SDM) is an increasingly important tool to predict the geographic distribution of species. Even though many problems associated with this method have been highlighted and solutions have been proposed, little has been done to increase comparability among studies. We reviewed recent publications applying SDMs and found that seventy nine percent failed to report methods that ensure comparability among studies, such as disclosing the maximum probability range produced by the models and reporting on the number of species occurrences used. We modeled six species of *Falco* from northern Europe and demonstrate that model results are altered by (1) spatial bias in species’ occurrence data, (2) differences in the geographic extent of the environmental data, and (3) the effects of transformation of model output to presence/absence data when applying thresholds. Depending on the modeling decisions, forecasts of the future geographic distribution of *Falco* ranged from range contraction in 80% of the species to no net loss in any species, with the best model predicting no net loss of habitat in Northern Europe. The fact that predictions of range changes in response to climate change in published studies may be influenced by decisions in the modeling process seriously hampers the possibility of making sound management recommendations. Thus, each of the decisions made in generating SDMs should be reported and evaluated to ensure conclusions and policies are based on the biology and ecology of the species being modeled.

## Introduction

Europe has the world’s most extensive network of conservation areas, which cover approximately 17% of the European Union’s surface [1]. However, climate change is expected to decrease the effectiveness of such areas to protect their biodiversity [2]. Indeed, climate change is already having effects in parts of Europe; the Arctic has, for instance, lost more than 2×10^6^ km^2^ of permanently frozen area in the last 1450 years [3], and this region hosts a large series of species specialized to circumpolar habitats [4]. In response to such effects, frameworks have been designed to assess the threats and benefits of climate change to species that focus on changes in their distributional ranges [5]. For example, multiple mammal and bird species in Mexico were modeled to predict these species’ future responses to climate change [6]. Another study characterized climate influences on the current distribution of endemic bird species of North America [7], and a recent study conducted a complete assessment of how many threatened species might be retained within the network of national versus the network of Natura 2000 conservation areas in Europe [2]. Many more such studies have been undertaken and are currently underway to help conserve biodiversity in the face of climate change. Predictions of how species’ distributions responds to changes in climate frequently use one of a suite of methods variously called species’ distribution modeling (SDMs), habitat modeling, or ecological niche modeling (ENM). These methods all have a similar purpose: to provide a geographical distribution of the environmental requirements of the species [8]. They all stem from Grinell’s idea that a species’ niche is closely related to the area in which the species is distributed [9].

In the past 20 years SDMs have increasingly been used as a tool to plan and design species’ conservation efforts. SDMs appear in the literature in increasing numbers each year (data obtained from ISI Web of Knowledge, Figure S1) and they are applied in new contexts, such as epidemiology [10], agronomy [11], and the study of invasive species [12]. Furthermore, hindcasting (i.e., projecting species’ geographic distribution backward in time) SDMs in combination with molecular phlyogeography is used to infer the role of climatic refugia for species. Likewise, when combined with molecular studies, SDMs can help elucidate the phylogeography [13] and description of the evolutionary paths [14] of species. The increasing number of publications using SDMs, and the diversification of fields in which this method is applied, appeal to a real need to create consensus and set standards on how model construction and results are reported to enhance interpretation and comparability among studies. SDMs can also be used to understand macroecological patterns. For example, forecasting predictions across multiple species provides a better understanding of the conservation value of geographic regions with regard to their future potential importance in protecting biodiversity [2]. Hence, SDMs are increasingly being used to aid decisions and the formulation of policies in such broad-reaching disciplines as conservation, pest control and the management of introduced species, as well as in human health issues [2], [15], [16]. On the other hand, hindcasting the distribution of large numbers of species provides information on which areas have acted as refugia in the past [17], [18].

Other critical and exciting applications of SDMs include comparing geographic range dynamics between species and investigating how ensembles of species may respond to climate change. For example, SDMs can be an important tool in predicting future geographic distributions of multiple, co-distributed species to project the fate of biodiversity in specific areas and to identify geographic locations with high conservation value. These results in turn can aid policy makers in managing biodiversity.

There is an increasing tendency to group individual SDMs to portray patterns across multiple species or whole taxonomic groups [6]. The rising need to generate predictions for groups of species makes it essential that models generated for different species can be both replicated and compared. More importantly, the process of generating SDMs should be standardized and clearly reported in publications so that attempts to compare models constructed for different taxa are not confounded by methodological or statistical artifacts, but reflect real ecological and evolutionary tolerances of species to their climatic niches [19], [20].

Species’ distributions are largely determined by environmental variables, such as climate, trophic interactions and dispersal limitation, and the relative importance of these factors are likely to vary depending on the scale at which species’ distributions are modeled [7], [21]. Another important issue to address in SDMs is sample size; since all niche models require occurrence data, there must be careful quality control based on basic knowledge of the geographic range and biology of the organisms. Previous authors have demonstrated how using different sets of occurrence data render different results [22]. Further, the extent of the geographic region used to train the model is also of primary importance, since the algorithms rely on background conditions to contrast with conditions at species’ occurrences and absences, and different results are obtained when using larger or tailored geographical extents [23]. Moreover, the AUC values that are currently regarded as a standard method for assessing the model performance are subject to large errors. Hence, AUC values are not a reliable method to assess model performance [24]. In addition, to demonstrate the lack of standardization in SDMs, a recent review of hindcasting studies found that four studies modeling climatic refugia in the Amazon basin each resulted in different predictions [25]. As each of these studies made different decisions when projecting species’ distribution to past climatic conditions, little consensus could be reached on which regions of the Amazon were predicted to be refugial areas, not just for one taxa, but for biodiversity in general. Since the idiosyncrasies and consequences of hindcasting and forecasting species’ geographic distributions are similar, a lack of consensus over which areas should have high priority for conserving future biodiversity is also plausible. The use of hindcasting species’ geographic distributions to validate evolutionary paths of speciation have been successfully applied in studies investigating a small set of refugia, based on phylogeographical studies such as speciation in the thrush-like mourner [13]. However, it is still uncertain whether the climatic refugia inferred are relevant also for other taxa or other hindcasting studies conducted in the same region.

Here, we first review recent publications applying SDMs and assess whether they provide the relevant information needed to ensure comparability among the predictions of the studies. This assessment is based on whether the publication provides information on how occurrence locations were handled and reported, the geographical extent of the region studied (both for training and projecting the model), the type of thresholds used to transform the continuous prediction to a binary one and how the accuracy and precision of the prediction was validated.

Second, we modeled the breeding distribution of six species of *Falco* in northern Europe and examined how model results were influenced by (1) spatial bias in species’ occurrence data, (2) differences in the geographic extent of the region studied, and (3) the effects of transformation (or thresholding) of a continuous model output to presence/absence data applying thresholds. We chose to model the breeding range of species within the *Falco* genus because large birds of prey are predicted to negatively respond to climate change since key natural history traits such as egg laying and clutch size have been correlated with North Atlantic climatic oscillations [4], [26].

## Materials and Methods

### Literature Search

**Figure 1:**
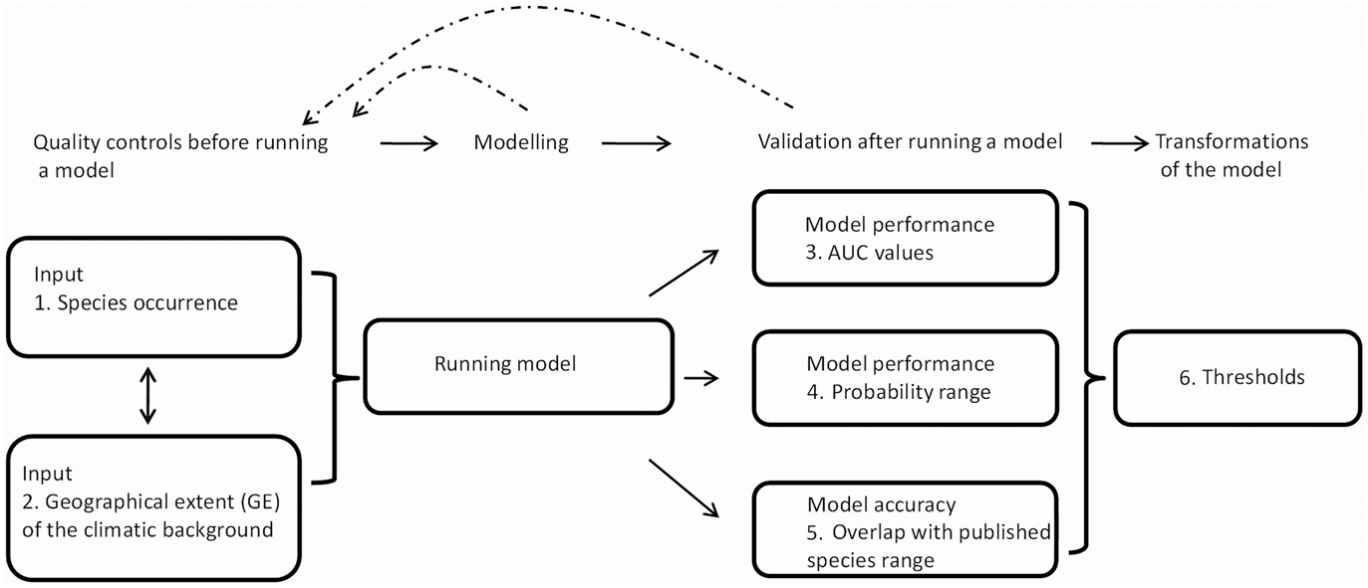
Schematic framework for generating, validating and processing SDMs for studying effects of climate on biodiversity.

As SDMs are often referred to by other names, we performed a literature search that considered both SDMs and ENMs. The term SDMs was most commonly used in the literature; for example, a search in the ISI web of knowledge on “species’ distribution modeling for 2010–2011 and refined by the query “MaxEnt or GARP or Artificial Neural Networks”, popular machine learning methods to model species distributions, returned 271 hits. Refining this query by “ecological niche modeling” resulted into 91 hits. When we searched for “ecological niche modeling” limited to the years 2010–2011 and refined by the query “MaxEnt or GARP or Artificial Neural Networks” first, there were only 97 hits. Refining this query by “species distribution modeling” resulted into 91 hits again. Therefore, 94% of the papers overlapped while searching for ecological niche modeling or species distribution modeling, and the latter returned a larger number of papers. Since the use of “species’ distribution modeling” returned a larger number of papers, we focused our subsequent literature search on publications that had “species’ distribution modeling” either in the title, keyword or abstract. We first counted how many recent publications employed SDMs by conducting a search in the Web of Science (http://apps.webofknowledge.com/) for the period of 1992–2011, using “species’ distribution model*” as a search term and extracted data to study trends in the use of this method during the past 20 years in order to evaluate how SDMs are applied and reported. We then selected the 317 studies that published on SDMs during the past two years (i.e., 2010–2011). We further restricted our analysis to studies that applied machine learning models such as MaxEnt or GARP, resulting in 170 publications. From these, we randomly sampled about half of the publications (77 publications), which we deemed a sample size sufficient for the purpose of our study. We recorded whether SDMs assessed one or multiple species and examined whether one could replicate SDM constructions based on the 9 criteria outlined below. We examined the selected publications to see if each reported (1) the number of species’ occurrences, (2) actions taken to resolve possible biases in occurrence data, such as lack of or too many occurrences in specific areas, (3) model evaluation by splitting data into testing and training data, (4) the explicit geographic extent of the region studied (i.e., we deemed it insufficient to present only a map of the study area; we required a statement on how the region from which climatic variables were drawn in order to predict species’ distributions was delimited [27], (5) the modeling algorithm(s) used, (6) the maximum probability of the resulting model, (7) the application of (a) threshold(s) to the continuous probability surface to create binary presence/absence data, (8) the type of precision test(s) employed, and (9) steps taken to test the accuracy of the predictions. (For definitions of terms applied here, see Table S1). We then calculated the proportion of studies that met each of the criteria. Moreover, we tallied the proportion of studies that reported and addressed the sources of error in their SDMs based upon the steps displayed in Figure 1.

### Case Study

Next, we tested how the predicted (future) breeding season ranges of six bird species in the genus *Falco* in northern Europe were influenced by decisions taken in modeling their geographic distributions. We focused on alternative decisions regarding (1) the input of species’ occurrence data, (2) the selection of the geographical extent of the environmental data, and (3) the effects of transformation of model output when applying thresholds (Figure 1). We then investigated how these decisions affected (1) the area under the curve (AUC) of ROC plots [28], (2) the maximum probability of the predicted suitable area, and (3) the accuracy of the predictions when compared to published ranges (Figure 1). Although there are numerous additional issues involved in building SDMs, we chose to focus on these major decisions and their consequences with regard to the reliability of predictions, since these steps most likely affect how results from a published SDM can be reviewed and its applicability assessed [19], [20]. We used MaxEnt [29] to illustrate and discuss the comparability of SDMs, but the issues discussed here are also applicable to other modeling algorithms. We chose MaxEnt because it has excelled compared to other algorithms in predicting species’ distributions that reflect the true physiological or mechanistic constraints of species to climatic conditions [30]. Furthermore, it performs better than other models such as BIOCLIM and GARP in situations where true absence data are unavailable [31], [32], and MaxEnt curently ranks among the most popular methods to construct SDMs.

We included nineteen bioclimatic variables derived from recent (1950−2000) monthly temperature and rainfall records described and available at WorldClim (http://worldclim.org/futdown.htm, [33]). Although the use of multiple auto-correlated bioclimatic variables is debated among species’ distribution modelers [22], [31], we included all 19 climatic variables, taking advantage of the regularization application in MaxEnt [30]. Regularization deals with the selection of environmental variables (regulating some to zero) and has performed well or even outperformed other modeling procedures that pre-select variables [34]. Furthermore, MaxEnt minimizes autocorrelation between variables, as it gives more weight to variables exhibiting high correlation with the occurrence data [35]. We used the default convergence threshold (10^−6^) and number of iterations (500). Hinge features were applied, as recommended by a comprehensive evaluation of MaxEnt [36]. The future climate projection was taken from the general circulation modelCGCM2 for 2080 downscaled to 30 arc-seconds, under emission scenario A2 (http://www.worldclim.org/futdown.htm).

### Model Construction

#### Species’ occurrence data

**Figure 2:**
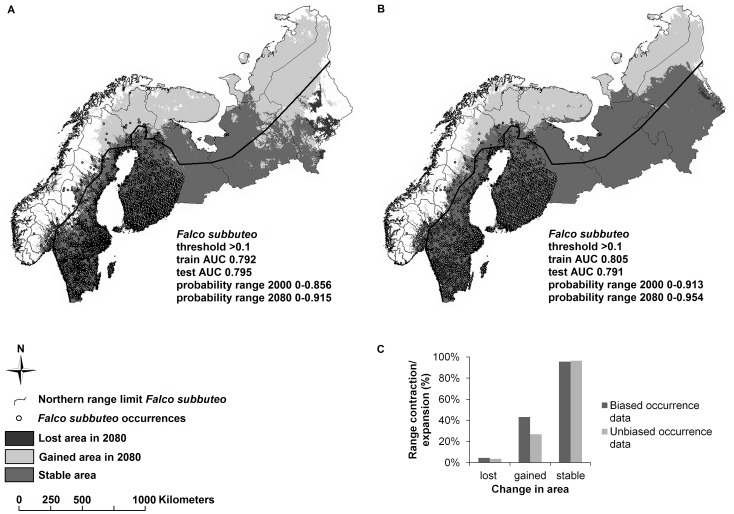
Example on how occurrence data can alter predictions of SDMs. **A**. Biased occurrence, versus **B**. Unbiased occurrence data affect the present and future SDM projections for *Falco subbuteo*, summarized in panel **C**.

We collected breeding season (June – August) occurrence data from 2000–2010 for six *Falco* species that occur in northern Europe from national and global databases (http://www.artsobservasjoner.no, http://www.artportalen.se, http://www.hatikka.fi, and http://data.gbif.org). By means of randomized partition, 30% of the occurrence data were set aside as testing data to validate the model. The remaining localities were used to train the model. To test how biased or under-sampled occurrence data affected MaxEnt models, we first modeled species’ distributions with all available data points (i.e., biased set because some locations have clumped occurrences whereas other locations have few occurrences [Figure 2]). We then modeled species’ distributions after minimizing potential bias in the occurrence data. To correct for clustered occurrence records that affect SDM predictions [22], we used a raster grid with a resolution of 10 arc-minutes and randomly selected one record per cell in order to reduce the bias potentially introduced by differences in human monitoring effort (i.e., unbiased set). Since *Falco* occurrence records were unavailable in north-western Russia, we projected predictions from Finland, Sweden and Norway to the parts of north-western Russia within our target region. The species modeled and numbers of occurrences included in both datasets were: *F. columbarius* (N_biased = _1691, N_unbiased = _1249), *F. peregrinus* (N_biased = _618, N_unbiased = _371), *F. rusticolus* (N_biased = _94, N_unbiased = _84), *F. subbuteo* (N_biased = _4644, N_unbiased = _1902), *F. tinnunculus* (N_biased = _5913, N_unbiased = _2689), and *F. vespertinus* (N_biased = _195, N_unbiased = _169).

#### Geographic extent of the region modeled

**Figure 3:**
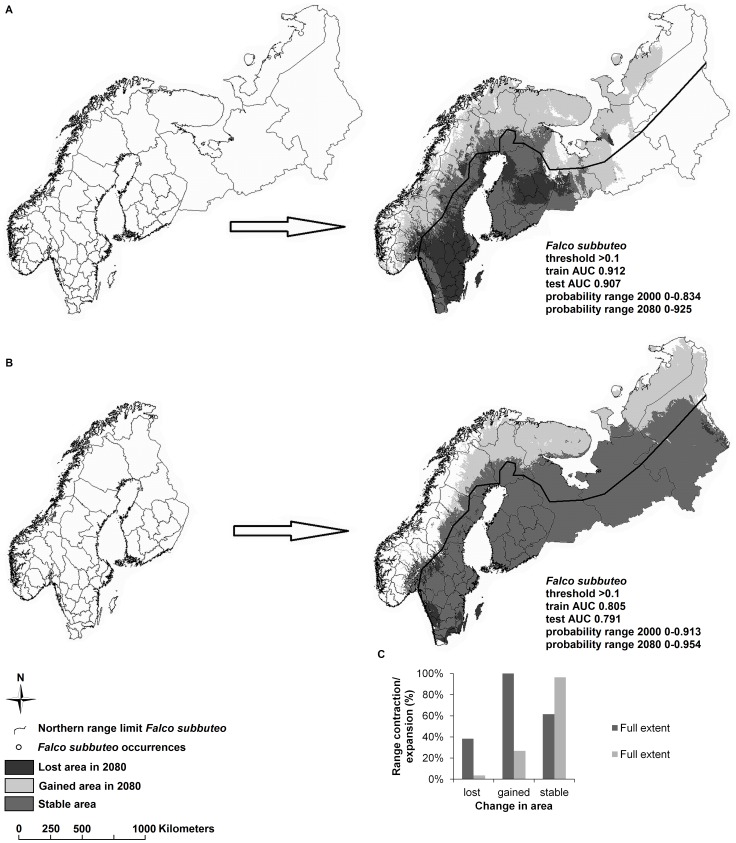
Example on how geographic extents impact conclusions made in SDMs of *Falco subbuteo.* **A**. Unrestricted extent versus, **B**. Restricted extent, and **C**. Results drawn from SDMs.

To study the effect of altering the geographic extent of the region modeled (i.e., the area in which the model is trained) on predicted species’ distributions, we first generated SDMs using a wide geographical extent including the entire region of Fennoscandia and north-western Russia (map shown in Figure 3B). Due to the paucity of *Falco* records in north-western Russia, using a full geographic extent of the region modeled may characterize the realized distribution of species in the genus poorly. Including large areas increases the chance that the model samples pseudo-absences in areas that have suitable conditions for the species but are falsely classified as unsuitable because the species has not been properly sampled in that region [8]. Indeed, choosing the correct extent is not a trivial task since the values where occurrence data are lacking are taken as pseudo-absences that are meant to provide a comparative data set to establish the conditions where a species may occur. If large extents with great environmental variation are selected, predictive models will be dominated by parameters that serve to coarsely discriminate regional conditions and weaken the ability to tease out fine-scale conditions determining presence or absence of species [27]. On the other hand, using a restricted region for selection of pseudo-absences can be a serious error when fitting models to project potential effects of climate change [37], since future environmental conditions may not be represented. Since occurrence data for our model species was lacking for north-western Russia, we used Fennoscandia, which accurately mirrored the distribution of the occurrence data of the species (map shown in Figure 3A).

#### Probability range

***First, we evaluated the effects of decisions made during model*** construction by investigating how locality data and geographical extent can affect the probability range of the predicted suitability of conditions, specifically the maximum probability generated by the models. The probability range is represented as a continuous surface of similarity values from low (0) to high (∼1) similarity, with high similarity values best representing environmental conditions similar to the conditions at the species’ known localities [29].

#### Thresholds

Once predictions were generated, we converted the probability range of projected conditions to a binary presence/absence surface by setting thresholds. A threshold is set by defining the probability value above which environmental conditions are deemed suitable for the presence of a particular species, and below which environmental conditions are considered unsuitable. In many applications, such as estimations of species’ range dynamics with changing climate, setting thresholds is a critical step in how model outcomes are interpreted. The selection of a threshold value is especially complicated without true absence data providing information on conditions under which the species is unlikely to occur. Here, we do not discuss the best approach in choosing a threshold, but present a few strategies for setting a threshold to assess how different thresholds can affect ecological conclusions. A range of approaches can be used to determine which threshold is most suitable [22], [38], [39]; one common approach is to set threshold values based on receiver operating characteristic (ROC) plots [38]. Model predictions in the ROC plots of *Falco* suggested that low threshold values may be appropriate, and we used one generous (0.1) and one more conservative (0.4) threshold value and compared the results.

### Model Evaluation

#### Probability range

The maximum probability given by a SDM is a good way to evaluate the effects of decisions made during model construction, since model predictions with high maximum probability (close to 1) reflect a good correlation between the variables included in the model and the species’ occurrence data used as input.

#### AUC values and overlap with published geographic distributions of species

Until recently, the area under the curve (AUC) of a ROC plot [20] was commonly used as the single measure to validate the predictions generated by SDMs. However, concerns have risen with regard to the usefulness of the metric [24], [40]. Still, since the vast majority of studies continue to rely solely on AUC values to validate the quality of models (see below), we decided to state the values of both the training and testing data and discuss how different decisions with regard to the input of the models affected the AUC values. For a thorough discussion on the usefulness of AUC values, we refer to papers dealing with this matter [25], [40] A practical approach to validate models is to use a hierarchical fuzzy pattern-matching approach to compare predictions generated by SDMs with published range maps [22]. This approach is not optimal since it assumes that published range maps are accurately depicting the distribution of a species, which might not be true. Furthermore, SDMs predict the potential niche and not the realized niche of a species. Below we discuss the limitations of this method more in depth, but due to the lack of an un-criticized approach to validate models available, we compared the predictions of the ranges generated by SDMs for current climatic conditions with published geographic ranges of European birds [41]. We expressed the similarity between predicted and published range maps using the percentage of the predicted current range that lay within the published range and the percentage of the published range that was covered by the predicted current range. In order to test the relationship between AUC values and overlap between published ranges and the SDMs we generated, we correlated AUC and percent overlap using Spearman’s rho (PASW statistics v. 18).

## Results

The use of SDMs has increased non-linearly over the last decade (Figure S1). A sampling of recent publications (2010–2011; Table S2) revealed that SDMs are now commonly used as a tool in applied ecology (i.e., 80% of the reviewed SDM studies), such as to inform decisions and direct policies in biodiversity conservation, management of introduced species and pest control (Figure S2A). Twenty-one percent of the investigated studies provided the information necessary to replicate their models along with information on how the models were evaluated (Figure S2B), whereas in the remaining 79% of the studies, one or more of our criteria for replication and evaluation were not met. The most neglected criteria were, reports on possible biases in species’ occurrence data (39%),and the maximum probability range produced by the models, with only 21% of the studies reporting on it(Figure S2B). In contrast, some criteria are more frequently reported; 83% of studies stated how many occurrence records were included in models, and 94% reported the use of a measurement to assess model performance in terms of precision, with 78% relying solely on AUC values (Figure S2B).

**Figure 4:**
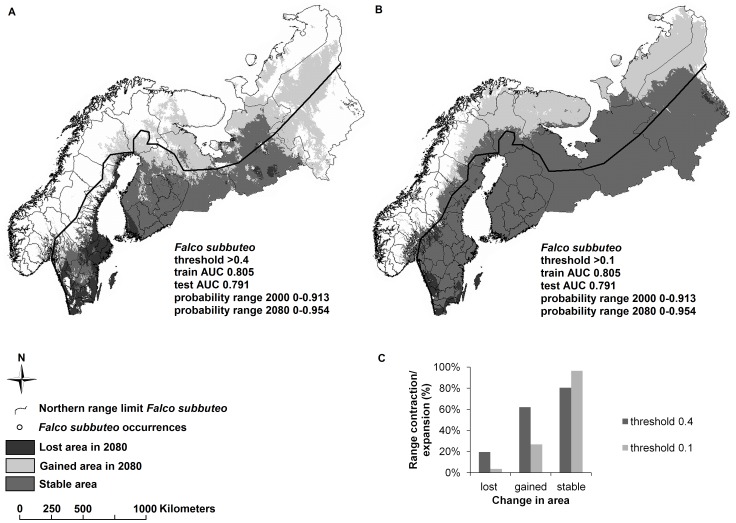
Example on how threshold choice for distribution probabilities impact predictions of SDMs of *Falco subbuteo.* A. Threshold greater than 0.4, B. Threshold greater than 0.1, C. the estimates of lost, gained and stable (refugial) areas of *Falco subbuteo.*

Table 1 illustrates the effects of making different decisions in SDM construction on predicting future geographic distributions for species in the genus *Falco*. Combined, the different decisions resulted in predictions ranging from reduced future range sizes in five out of six species to no reduction in range size for any of the six species. Graphical examples of the impact of occurrence records, geographic extents and thresholds on the predicted breeding range of the Eurasian hobby (*Falco subbuteo*) are given in Figures 2 – 4. Using biased locality data, in which occurrences were clumped, led to altered conclusions regarding the number of species predicted to experience a future range contraction or expansion. In general, applying biased occurrence data to SDMs resulted in poorer predictions, i.e., decreased similarity between predicted current ranges and published ranges and lower AUC values in the training data (Table 1, Figure 2). Moreover, using biased data produced more generous estimates of mean area gained in the future than applying unbiased occurrence data. In addition, current and future predictions for *Falco* species showed large differences in predicted suitable breeding ranges in northern Europe when we altered the geographic extent of the region modeled (Table 1, Figure 3). Predicted suitable areas generally increased and became more similar to published ranges when a restricted extent (Figure 3) and lower thresholds (Figure 4) were applied. Restricting the geographic extent generally increased the maximum probability range but lowered the mean of the AUC values (Table 1). Thus unbiased occurrence records, restricted geographic extents, and low thresholds produced predictions that resembled most closely the published species’ ranges. However, these SDMs did not have the highest AUC values (Table 1). Indeed, AUC values of both training and test data were significantly negatively correlated with similarity between predicted current and published ranges (Spearman’s rho; train data: *r = *−0.82, *n* = 48, *p*<0.001; test data: *r* = −0.78, *n* = 48, *p*<0.001). As such, AUC values were high for SDMs with predictions that poorly reflected published ranges.

**Table 1:** Mean effects of decisions made in SDM construction on six *Falco* species.

Occurrence data	Extent of region modeled	Threshold		Maximumprobability range	Train AUC	Test AUC	Similarity	Change in area (%)	Trend
Biased	Full	0.1	mean	0.847	0.913	0.905	42%	107%	Four losers
			*se*	*0.042*	*0.028*	*0.025*	*11%*	*29%*	
Biased	Full	0.4	mean	0.847	0.913	0.905	37%	100%	Three losers
			*se*	*0.042*	*0.028*	*0.025*	*11%*	*19%*	
Biased	Restricted	0.1	mean	0.964	0.834	0.812	66%	115%	One loser
			*se*	*0.022*	*0.038*	*0.034*	*10%*	*18%*	
Biased	Restricted	0.4	mean	0.964	0.834	0.812	54%	173%	Two losers
			*se*	*0.022*	*0.038*	*0.034*	*9%*	*37%*	
Unbiased	Full	0.1	mean	0.904	0.934	0.917	44%	112%	Two losers
			*se*	*0.038*	*0.017*	*0.016*	*10%*	*23%*	
Unbiased	Full	0.4	mean	0.904	0.934	0.917	37%	86%	Five losers
			*se*	*0.038*	*0.017*	*0.016*	*11%*	*25%*	
**Unbiased**	**Restricted**	**0.1**	**mean**	**0.959**	**0.828**	**0.772**	**76%**	**118%**	**No losers**
			***se***	***0.021***	***0.038***	***0.033***	***8%***	***5%***	
Unbiased	Restricted	0.4	mean	0.959	0.828	0.772	58%	142%	No losers
			*se*	*0.021*	*0.038*	*0.033*	*9%*	*17%*	

The bold option highlights the best criteria applied to the study. Similarity is expressed in the average between the percentage of the predicted current range that lay within the published range, and the percentage of the published range that was covered by the predicted current range. The ‘percentage gained area’ and ‘trend’ are based upon the difference between current and future prediction.

## Discussion

### Decisions Altering Model Outcomes

We have demonstrated how decisions made while generating SDMs can often significantly alter modeling outcomes. For example, applying unbiased occurrence data to a restricted extent of the region modeled in our *Falco* models improved the similarity between predicted current and published ranges (Table 1). Changing these criteria also influenced the maximum probability of area suitability in the generated predictions, and thresholding with different values across the probability surfaces modified predicted range sizes. These decisions had far reaching consequences in terms of conclusions that could be drawn from the modeling effort. Compared to what we deemed the best modeling strategy according to model evaluation, the conclusion regarding future range expansion or contraction changed in five of six species (Table 1). Although we do not know if the decisions made during the modeling process affect predictions for *Falco* more or less than they would any other genus, absence of such influence can only be guaranteed by reporting the decisions made. Yet despite the evident impacts made with each choice in designing SDMs, less than half of the published studies we sampled reported their methods in a way that facilitated repeatability and comparability to other SDMs. Failure to report on such variables as bias in locality data, geographic extent of climatic layers, values of resulting maximum probability surfaces or thresholds imposed on those surfaces significantly hinders any generalizations that could be made when comparing models across taxa [25].

The model with highest support for the accuracy and precision tests suggests no *Falco* species will lose habitat (Table 1). Decisions in modeling that improved the accuracy and precision of our predictions were: 1. using unbiased data, 2. using a restricted extent of the region modeled, and 3. using a generous threshold. An even distribution of occurrences across the model is important to ensure that the model will not rank conditions of a site as more suitable because it is better known or has higher number of data records [22]. A different source of error, which should be addressed before modeling, is the imprecision of GIS coordinates in the occurrence data; this error is accentuated when the number of occurrences available is small. Previous studies have shown that boosted regression trees and MaxEnt are less influenced by these types of error, [42]; however a recent study demonstrated that for other species, GARP was less influenced, supporting the use of a variety of modeling techniques as opposed to a single one [43]. Another important factor was to calibrate the models for regions with good data availability (i.e., using a restricted extent of the region modeled, which in our study meant excluding north-western Russia from input in the models and then projecting to it) instead of using large extents of suitable conditions for which poor occurrence data exists [8], [23]. However, if the geographic extent of the region modelled is too small, the breadth of the environmental conditions used to generate predictions may not capture the full environmental niche of the species. This might also significantly influence predictions [37]. We therefore recommend considering the boundaries of the geographic extent used in models carefully, and it is likely optimal to use a geographic extent that captures as much as possible from the environmental niche of the species as long as there is no geographical bias in the occurrence data.

Choosing a threshold is not a trivial step in species’ distribution modeling and there is not a “magic” value for thresholding models, since the continuous probability range may not extend to 0.999. Secondly, the best threshold to use depends on the quality of the model. Choosing thresholds that maximize the resemblance of predicted current ranges with published ranges and contrast with known physiological boundaries of the species may be a more biologically meaningful method for choosing thresholds [39], [44]. However, factors that are generally not included in models, such as dispersal barriers, competitors and anthropogenic factors, might constrain species’ distributions [45]. Thresholding to maximize the resemblance of predicted current ranges with published ranges might bias the results when projecting to the future, because the full breath of the environmental niche of the species might not be captured. Ideally, non-climatic variables that may constrain species ranges should [46], and could, to a limited extent [15], be included in models. When this is not feasible, it may be argued that applying a threshold may substitute or account for constraints to species’ ranges set by non-climatic variables, to a certain extent.

We found a negative correlation between AUC values and the accuracy of the predicted current geographic ranges; which implies that models with lower AUC values produced current geographic ranges that were more in agreement with published ranges. A possible cause of this counterintuitive finding might be that the approach to validate SDMS by comparing generated predictions with published ranges is invalid, as is further discussed below. It however might also suggest that AUC values are not useful for the evaluation of SDMs, or that there might be fundamental problems within the model (e.g. pseudo-absences might not closely represent true absences). A thorough investigation might clarify this. Nevertheless, previous findings show that AUC values may indeed mislead readers to think that models are accurate in predicting the distribution of species [24], [47]. Despite these findings, our literature review showed that a solid majority (75%) of studies employed only AUC values to assess model accuracy. Using ROC curves and AUC values could greatly be improved by adding pseudoabsences or target group absences, which are locations experts have surveyed for specific species and reported their absence [48]. However, these data are not available for large numbers of species and expert knowledge is usually difficult to implement when studying multiple taxa. Perhaps in addition to reporting AUC values, models may be further validated by comparing predicted current ranges with published ranges [19] as we have done, assuming reliability of the latter. Comparing the overlap of current predictions with published ranges is one of the multiple suboptimal methods available to compare the performance of models. One of the major problems with this approach is that it assumes that the published range is accurate, which may often not be the case. Single species often have multiple published ranges that may disagree with one another and provide no information on what presentation is more accurate. Published ranges are also not available for many species. Furthermore, the ecological niche of a species may be conservative with low environmental tolerance, but this does not preclude the possibility of adaptation to new conditions [49]. The current distribution of a species may also be constrained or enabled by, e.g., anthropogenic factors, natural barriers or by biotic interactions with other species [15]. Ideally, predictions of a species’ geographic distribution should be compared with known limits in fitness and tolerances to the environmental conditions that restrain its niche [44] and the biotic conditions, like mutualisms and predation, which enable/prevent local extinctions of populations at range limits. However, such information is available for few species. Besides, whilst published ranges are assumed to show realized ranges of species, SDMs depict potential ranges; this fundamental difference might be highly problematic for accurate validation of predictions. Thus, the approach of comparing the overlap between the model output and a published range is what we deem a feasible, indicative (if yet somewhat limited) method for model evaluation. Other authors concur that the comprehensive knowledge of experts who published species’ distribution maps is an acceptable form of confirming species’ distribution models, especially since a better alternative is unavailable at present [44].

### Management Implications

Predicting the risk for future range contractions/expansions of species as a result of climate change is one of the most common aims of SDMs. If results from SDMs are not shown to be immune to influences from decisions made during model generation, they should not be used as a sole basis for management recommendations to protect species. Our results also have implications for prioritization of areas for conservation. Current efforts intend to identify areas of future conservation interest by identifying regions where the most species are predicted to occur using the geographic overlap from multiple SDMs. However, we demonstrated how the overlap of species’ SDMs can range from a maximum to a minimum level depending on the decisions taken to generate the models. These results affect decisions and the design of migration corridors and future viability of nature reserves. Thus, in order to improve predictions that aid management and planning of nature reserves, one must carefully determine each step of the SDM model and ensure that the prediction of the geographic distribution of a species is based on its biological constraints. Similarly, if SDMs are used to predict areas vulnerable to invasion of species, care must be taken to ensure that the SDM reflects the key environmental and biological variables that may enable or restrict the distribution of the invasive species.

### Conclusions

In conclusion, since the approaches to generating and evaluating SDMs affect model predictions, which can have far reaching impacts for policy decisions such as in species’ conservation, we argue that modelers should consistently report the criteria they used (e.g., geographic extent of climate layers, thresholding values) and the limitations of the resulting models (e.g., maximum probability values). Our study does not aim to provide a ‘recipe’ for constructing SDMs. Rather, we have demonstrated that decisions made while generating and transforming predictions of species’ distributions affect the quality and accuracy of the resultant models and their applications. Therefore, selection of input data and model parameters should be deliberated carefully so as to optimize model performance and ecological applicability. Reporting of these parameters will also allow for greater comparability and applicability among SDMs. For SDMs to fulfill their role as an important tool in ecology, authors of scientific papers, as well as journal editors and reviewers need to raise the standards regarding the information on modeling procedure and evaluation that needs to be disclosed when reporting results of SDM efforts.

## Supporting Information

Figure S1 **Number of studies using SDMs as listed in the Web of Knowledge from 1992–2010.**
(TIF)

Figure S2 **The analyses of a subsample of papers using SDM. A**. Layout of the published ecological applications in which SDMs are used. **B**. Proportion of SDM publications reporting on species’ occurrences, bias in the input data, geographical extent, maximum probability distribution, thresholds used to transform continuous probability surfaces to binary surfaces, tests of SDM precision, and tests of SDM accuracy.
(TIF)

Table S1 **Definitions of terms we used in species’ distribution modeling.**
(DOC)

Table S2 **Individual studies analyzed.**
(DOC)
