# Supplementary figures and images for: Predicting the Fate of Biodiversity Using Species’ Distribution Models: Enhancing Model Comparability and Repeatability
Source: PLoS One. 2012 Sep 11;7(9):e44402. doi: 10.1371/journal.pone.0044402 (PMC3439421; doi:10.1371/journal.pone.0044402)

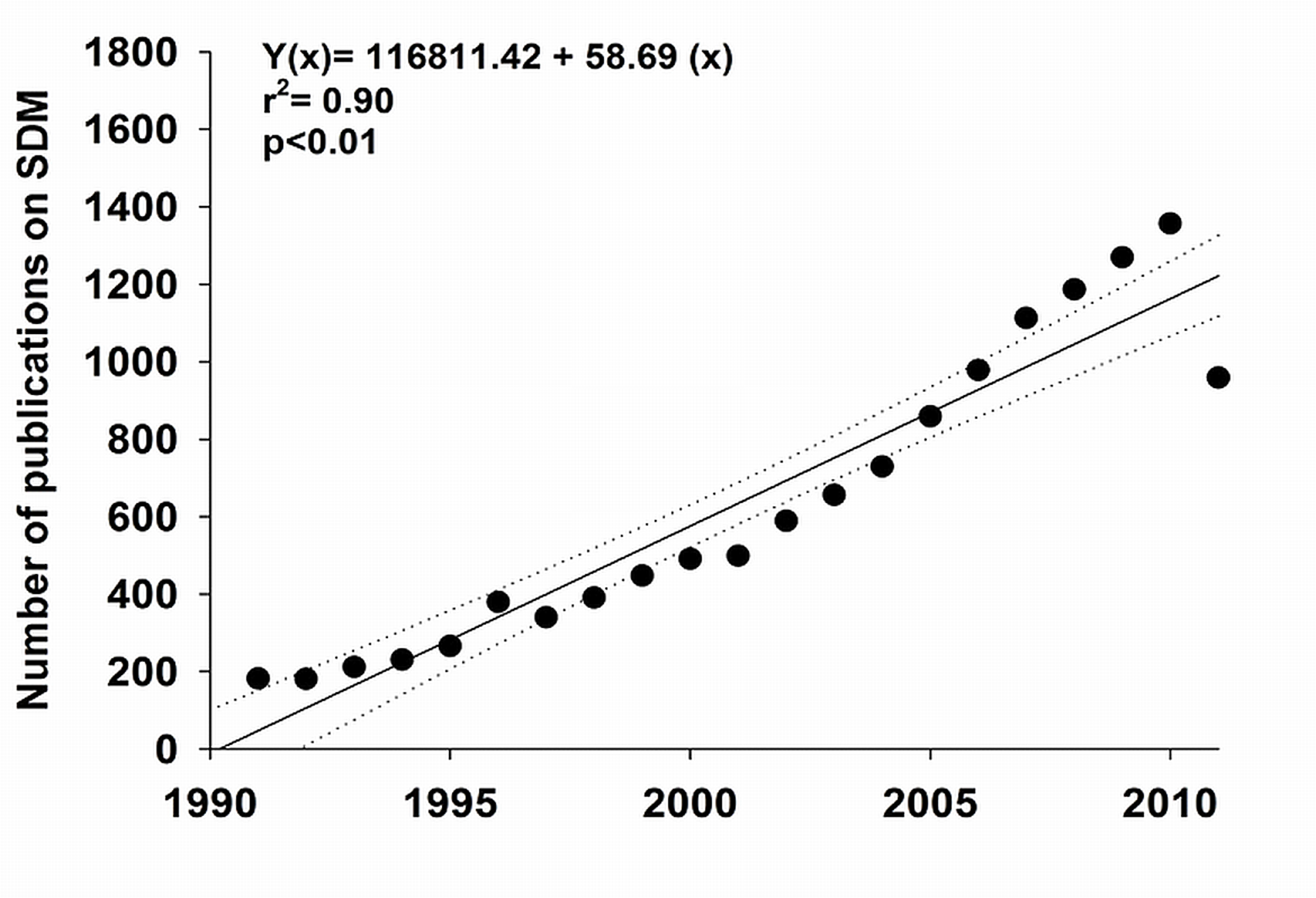

Supplement: Figure S1 — Number of studies using SDMs as listed in the Web of Knowledge from 1992–2010. (TIF) [file pone.0044402.s001.tif]

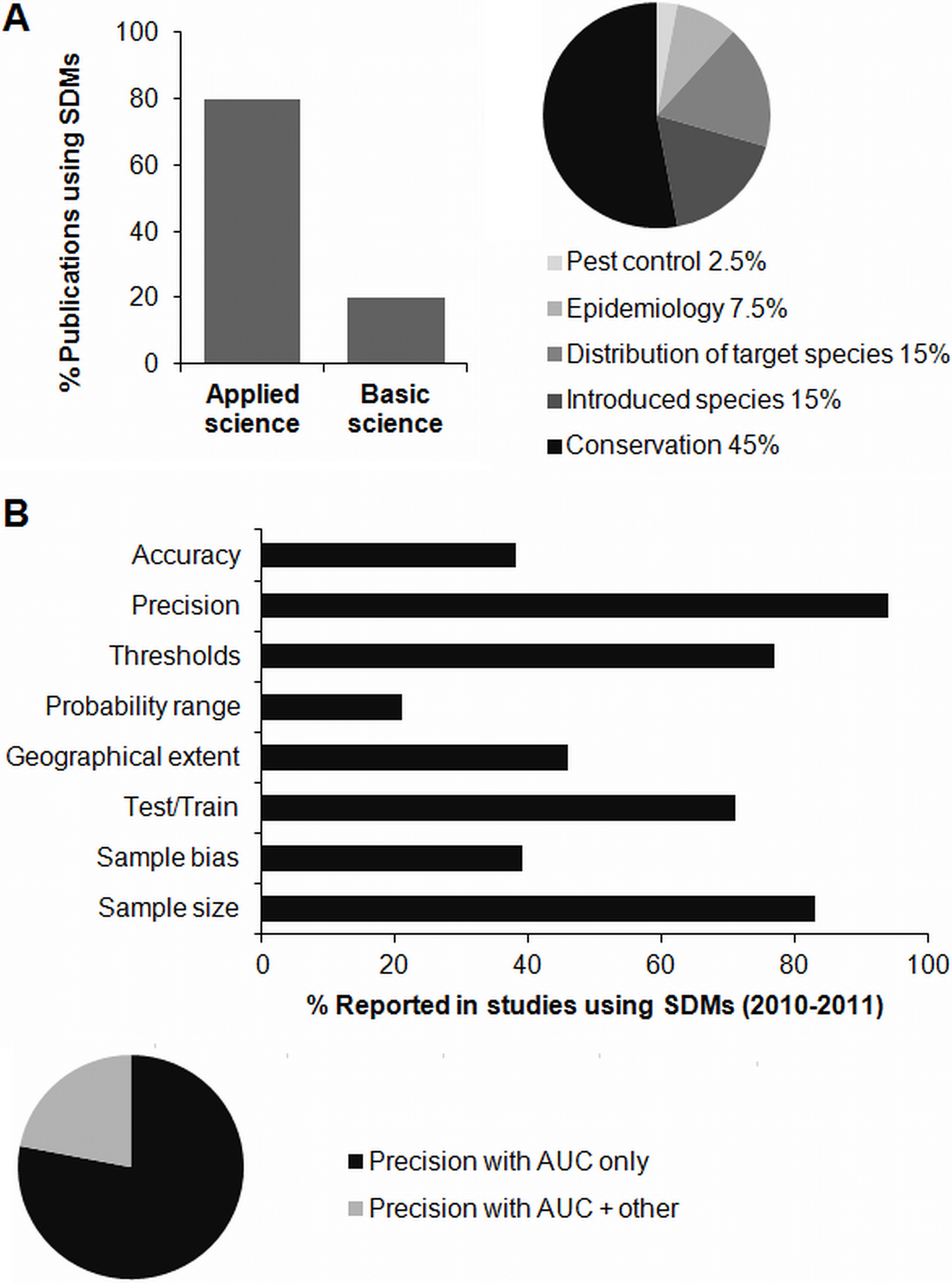

Supplement: Figure S2 — The analyses of a subsample of papers using SDM. A. Layout of the published ecological applications in which SDMs are used. B. Proportion of SDM publications reporting on species’ occurrences, bias in the input data, geographical extent, maximum probability distribution, thresholds used to transform continuous probability surfaces to binary surfaces, tests of SDM precision, and tests of SDM accuracy. (TIF) [file pone.0044402.s002.tif]
