# Supplementary material for: Predicting the Fate of Biodiversity Using Species’ Distribution Models: Enhancing Model Comparability and Repeatability
Source: PLoS One. 2012 Sep 11;7(9):e44402. doi: 10.1371/journal.pone.0044402 (PMC3439421; doi:10.1371/journal.pone.0044402)
Supplement: Table S1 — Definitions of terms we used in species’ distribution modeling. (DOC) [file pone.0044402.s003.doc]

## Table S1. Definitions of terms we used in species’ distribution modeling.

| **Definitions** | |
| --- | --- |
| Accuracy | The level with which the modeled output represents the true situation |
| AUC | Area Under the Curve of a ROC plot (Hanley and McNeil 1982) |
| Extent of the environmental variables | The geographical area for which environmental conditions are used as input variables in a SDM to generate a predictive output |
| Forecasting | Prediction into the future |
| Hindcasting | Prediction into the past |
| Losers | Species that acquire a smaller geographical distribution over time |
| Overfitting | The model describes random error, i.e. has a poor predictive performance |
| Overprediction | Prediction of presence in areas which do not contain the focal species |
| Probability range | The range (min. 0, max. 1) of the predicted probability of area suitability generated across geographical space. Higher numbers define better predicted conditions for the species to occur. |
| ROC curve | Receiver Operating Characteristic curve (Hanley and McNeil 1982) |
| Sensitivity | The fraction of true species presence values, i.e. the absence of omission error |
| Specificity | The fraction of false species presence values, i.e. the omission error |
| Test AUC | The AUC value of the 30% of occurrence data which were set aside as ‘test’ data |
| Threshold | Subjectively determined numerical cut-off above which a species is likely to be present |
| Train AUC | The AUC value of the 70% of occurrence data which were set aside as ‘train’ data |
| Underprediction | Observed occurrences of the species fall outside the modeled geographic range |
| Winners | Species that acquire a larger distribution range over time |
