# Supplementary material for: Predicting the Fate of Biodiversity Using Species’ Distribution Models: Enhancing Model Comparability and Repeatability
Source: PLoS One. 2012 Sep 11;7(9):e44402. doi: 10.1371/journal.pone.0044402 (PMC3439421; doi:10.1371/journal.pone.0044402)
Supplement: Table S2 — Individual studies analyzed. (DOC) [file pone.0044402.s004.doc]

**Table S2**. Individual studies analyzed.

| **Reference** | **Application** | **Single/multiple** | **Occurrence data (n)** | **Bias in the occurrence data** | **Test/Train** | | **Geographic extent** | | **Modeling algorithm** | | | **Probability distribution** | | | **Thresholds** | | | **Precision test** | | | **Accuracy test** | | | | | |
| --- | --- | --- | --- | --- | --- | --- | --- | --- | --- | --- | --- | --- | --- | --- | --- | --- | --- | --- | --- | --- | --- | --- | --- | --- | --- | --- |
| Achcroft et al 2011 | methods,climatic variables | multiple | R | R not solved | R | | NER | | MaxEnt | | | MNR | | | NA | | | AUC | | | ID crossvalidation | | | | | |
| Alba-Sanchez et al 2010 | spatial evolution | multiple | R | RS | R | | NR | | MaxEnt | | | R | | | NA | | | AUC* | | | ID correlations between environmental factorsX prob presence | | | | | |
| Aranda and Lobo 2011 | conservation, reserves | multiple | R | RS | NR | | NER | | MaxEnt | | | R | | | single | | | Actual errors in prediction | | | Modelled vs. real diversity indices | | | | | |
| Araujo et al 2011 | conservation, reserves | multiple | NR* | NR* | R | | tailored to a predetermined area | | mean predictions of GLM, GAM, BRT,ANN, FDA,SRE | | | NR | | | NA | | | AUC,TSS* | | | NR | | | | | |
| Barbet-Massin and Jiguet 2010 | conservation, endemics | single | NR* | NR | R | | tailored to a predetermined area | | CTA, ANN, GBM, RF* | | | NR | | | multiple | | | AUC, TSS | | | average of models | | | | | |
| Beatty and Provan 2011 | spatial evolution | multiple | R | NR | R | | NR | | MaxEnt | | | NR | | | single | | | AUC | | | NR | | | | | |
| Bedia et al 2011 | geographic range estimation | multiple | R | R as no bias | NR | | NR | | Cart, MaxEnt, MLR, ANN, MARS, SVm | | | R | | | single | | | Kappa , TSS | | | NR | | | | | |
| Beukema et al 2010 | spatial evolution | multiple | R | R not solved | R | | NER | | MaxEnt+essemble analysis (Araujo & New 2006) | | | NR | | | single | | | AUC | | | NR | | | | | |
| Bittner et al 2010 | conservation, reserves | multiple | NA | NA | R | | tailored to a predetermined area | | RF, GLM | | | NR | | | NA | | | AUC, intercept & slope of calibration curve | | | NR | | | | | |
| Blach-Overgaard 2010 | geographic range estimation | multiple | R | R as no bias | R | | NR | | MaxEnt | | | NA | | | NA | | | AUC | | | ID correlation of pres/pseudoabs and model predictions | | | | | |
| Bradley et al 2010 | exotic species invasion | multiple | NA | R not solved | | | NR | | Maxent & Mahalanobis distance | | | R | | | NR | | | AUC | | | NR | | | | | |
| Bromberg et al 2011 | exotic species invasion | single | R | NR | NR | | NR | | MaxEnt | | | MNR | | | multiple | | | NR | | | NR | | | | | |
| Buckley 2010 | geographic range estimation | multiple | NR | RS | NR | | NR | | MaxEnt | | | NR | | | NR | | | sensitivity/specificity | | | contrasted with mechanistic model | | | | | |
| Carranza et al. 2011 | geographic | multiple | R | NR | R | | NR | | MaxEnt | | | NR | | | single | | | NR | | | NR | | | | | |
| Carroll 2010 | conservation | single | NER | RS | NR | | NER | | MaxEnt | | | NR | | | single | | | AUC | | | Cross validation | | | | | |
| **Reference** | **Application** | **Single/multiple** | **Occurrence data (n)** | **Bias in the occurrence data** | **Test/Train** | **Geographic extent** | | **Modeling algorithm** | | | **Probability distribution** | | | **Thresholds** | | | **Precision test** | | | **Accuracy test** | | | | | |  |
| Carroll et al 2010 | conservation, reserves | multiple | R | RS | NR | | NER | | MaxEnt | | | MNR | | | NA | | | AUC | | | NR | | | |  | |
| Carvalho et al 2010 | conservation, reserves | multiple | R* | R as no bias | R | | NR | | MaxEnt | | | NR | | | NA | | | AUC | | | NR | | | |  | |
| Carvalho et al 2010 | conservation, endemics | multiple | NER | NR | R | | tailored to a predetermined area | | MaxEnt, GLM, GAM, CTE, ANN, GBM, RF, MDSm MARS | | | NR | | | single | | | AUC* | | | NR | | | |  | |
| Cianfrani et al 2011 | conservation, climate change | single | R | RS | R | | tailored to a predetermined area | | ANN, GAM, GML, FDA, MARS, MaxEnt | | | NR | | | single | | | NR | | | crossvalidation, 3300 random absences calibration of model , | | | |  | |
| Corbalan et al 2011 | conservation, reserves | multiple | R | NR | R | | tailored to a predetermined area | | MaxEnt | | | NR | | | NA | | | AUC | | | NR | | | |  | |
| Crawford and Hoagland 2010 | conservation, endemics | single | R | RS | R | | NR | | GLM, GAM, CART, RF, GBM, MaxEnt | | | MNR | | | NA | | | AUC* | | | AUC** | | | |  | |
| de Pous et al 2011 | conservation, reserves | multiple | R | NR | R | | NER | | MaxEnt | | | NR | | | single | | | AUC | | | NR | | | |  | |
| Diniz-Filho et al 2011 | conservation, climate change | single | R | NR | R | | tailored by species | | Bioclim, euclidean, MAH, GARP, MaxEnt | | | NR | | | multiple | | | AUC, TSS | | | NR | | | |  | |
| Dobrowski et al 2011 | conservation, climate change | multiple | R | RS | R | | NR | | GLM, GAM,RF, GBM | | | MNR | | | NA | | | AUC | | | ID cross validation, transferability between two time periods . | | | |  | |
| Evans et al. 2010 | habitat suitability | single | R | NR | NR | | tailored to a predetermined area | | MaxEnt; support vector machine | | | NR | | | NA | | | AUC; Kappa; rank correlation | | | NR | | | |  | |
| Falk & Mellert 2011 | forest management planning | single | R | NA | R | | tailored to a predetermined area | | BRT; GAM | | | NR | | | multiple | | | AUC | | | potential natural distribution | | | |  | |
| Feira & Faulkes 2011 | exotic species invasion | single | NR | NR | R | | tailored to a predetermined area | | MaxEnt | | | R | | | NA | | | AUC | | | NR | | | |  | |
| Fernandez et al. 2010 | geographic range estimation | multiple | R | NR | R | | NER | | MaxEnt | | | NR | | | NA | | | AUC | | | NR | | | |  | |
|  |  |  |  |  |  | |  | |  | | |  | | |  | | |  | | |  | | | |  | |
| **Reference** | **Application** | **Single/multiple** | **Occurrence data (n)** | **Bias in the occurrence data** | **Test/Train** | **Geographic extent** | | **Modeling algorithm** | | | **Probability distribution** | | | **Thresholds** | | | **Precision test** | | | **Accuracy test** | | | |  | | |
| Fischer et al. 2011 | exotic species invasion | multiple | R | RS | R | | tailored by species, | | MaxEnt | | | R | | | NA | | | AUC | | | NR |  | | | | |
| Fouquet et al. 2010 | conservation, species | multiple | R | RS | R | | Tailored by species | | MaxEnt | | | R | | | single | | | AUC; cross validation | | | NR |  | | | | |
| Gallagher et al. 2010 | exotic species invasion | multiple | R | NR | NR | | tailored to a predetermined area | | MaxEnt | | | NR | | | multiple | | | AUC | | | NR |  | | | | |
| Garzon et al. 2011 | spatial evolution | single | R | NR | R | | tailored to a predetermined area | | RF | | | NA | | | NA | | | AUC; variance explained | | | NR |  | | | | |
| Gibson et al. 2010 | conservation, climate change | single | R | NR | R | | Tailored by species | | MaxEnt | | | NR | | | single | | | AUC | | | ID |  | | | | |
| Gogol-Prokurat 2011 | habitat suitability | single | R | NR | R | | tailored to a predetermined area | | MaxEnt | | | NR | | | NA | | | AUC; Hosmer-Lemeshow goodness of fit; Min. acceptable error suitable area | | | ID |  | | | | |
| Graham et al. 2011 | exotic species invasion | single | R | NR | R | | tailored to a predetermined area | | MaxEnt | | | NR | | | single | | | AUC | | | NR |  | | | | |
| Habel et al. 2011 | conservation, climate change | single | R | RS | R | | NR | | MaxEnt | | | NR | | | multiple | | | AUC | | | NR |  | | | | |
| Habel et al. 2011 | spatial evolution | single | R | RS | R | | Tailored by species | | MaxEnt | | | NR | | | multiple | | | AUC | | | NR |  | | | | |
| Jackson & Robertson 2011 | geographic range estimation | single | R | RS | NR | | NER | | MaxEnt | | | NR | | | single | | | NR | | | ID |  | | | | |
| Jarnevich & Laubhan 2011 | conservation, species | multiple | R | NR | R | | tailored to a predetermined area | | MaxEnt | | | R | | | NA | | | AUC; Standard deviation of model runs | | | NR |  | | | | |
| Jiguet et al. 2010 | geographic range estimation | multiple | R | NR | R | | tailored to a predetermined area | | Ensemble of 8 methods | | | NR | | | single | | | jack knife | | | NR |  | | | | |
| Jimenez-Valvede et al. 2011 | exotic species invasion | single | R | NR | NR | | tailored to a predetermined area | | MaxEnt; GARP; DOMAIN; BIOCLIM | | | NR | | | single | | | NR | | | NR |  | | | | |
| Kuemmerle et al. 2011 | conservation, species | multiple | R | NR | NR | | NER | | MaxEnt | | | R | | | multiple | | | AUC; cross validation | | | NR |  | | | | |
| Larson | pest control | multiple | R | R not solved | R | | NR | | MaxEnt, GARP | | | NR | | | NR | | | AUC | | | NR |  | | | | |
| Liu | exotic species invasion | single | R | RS | R | | NR | | MaxEnt | | | NA | | | single | | | AUC, null models | | | NR |  | | | | |
| Lötters | methods | multiple | R | NR | R | | NR | | MaxEnt | | | NR | | | NR | | | AUC | | | NR |  | | | | |
| **Reference** | **Application** | **Single/multiple** | **Occurrence data (n)** | **Bias in the occurrence data** | **Test/Train** | | **Geographic extent** | | **Modeling algorithm** | | | **Probability distribution** | | | **Thresholds** | | | **Precision test** | | | **Accuracy test** |  | | | | |
| Marini | conservation | multiple | R | NR | R | | NR | | 9 sdms, amongst others MaxEnt | | | NR | | | multiple | | | AUC | | | NR |  | | | | |
| Marino | conservation | single | R | RS | R | | NR | | MaxEnt | | | NA | | | NR | | | AUC | | | NR |  | | | | |
| Martinez-Morales | conservation | single | R | NR | NA | | Tailored by species | | GARP | | | NA | | | NR | | | NR | | | NR |  | | | | |
| Milanovich | conservation | multiple | NR | RS | R | | NR | | MaxEnt | | | NR | | | multiple | | | AUC, null models | | | Prange |  | | | | |
| Morueta-Holme | conservation | single | R | NR | R | | NR | | MaxEnt, Bioclim | | | NA | | | classes | | | AUC | | | ID |  | | | | |
| Mukherjee | exotic species invasion | single | R | NR | R | | NR | | MaxEnt | | | NA | | | classes | | | AUC | | | NR |  | | | | |
| Murray | pest control | single | R | R not solved | R | | NR | | MaxEnt | | | NA | | | NR | | | AUC | | | NR |  | | | | |
| Nakazato | other | multiple | NR | NR | R | | NR | | MaxEnt | | | NR | | | NR | | | AUC | | | NR |  | | | | |
| Nobrega | conservation | multiple | NR | NR | NR | | NR | | MaxEnt | | | NR | | | multiple | | | AUC | | | NR |  | | | | |
| Nunez | exotic species invasion | multiple | NR | RS | NR | | NR | | MaxEnt | | | NR | | | NR | | | AUC | | | ID crossvalidation |  | | | | |
| Ogawa-Onishi | conservation | multiple | NR | NR | R | | tailored to a predetermined area | | GAM, ANN, GBM, RF | | | NR | | | multiple | | | AUC | | | NR |  | | | | |
| Olah-Hemmings | conservation | multiple | R | NR | R | | Tailored by species | | MaxEnt | | | NR | | | multiple | | | AUC | | | NR |  | | | | |
| Pittman | conservation | multiple | R | NA | NR | | tailored to a predetermined area | | MaxEnt, BRT | | | NR | | | single | | | AUC | | | ID crossvalidation |  | | | | |
| Politis | conservation | multiple | R | NR | R | | NR | | MaxEnt | | | NR | | | multiple | | | AUC | | | NR |  | | | | |
| Pompe et al. 2010 | conservation, climate & land-use change | multiple | NA | NA | NR | | tailored to a predetermined area | | variety, Random Forest | | | NR | | | maximize kappa | | | Kappa | | | NR |  | | | | |
| Ponce et al. 2011 | geographic range estimation | multiple | R | RS | R | | NER | | MaxEnt and API * | | | R | | | multiple | | | Wilcoxon-Mann-Whitney robust test, Mee's statistic | | | compared to lithologic and veg coverages, groundthruthing |  | | | | |
| Ready et al. 2010 | geographic range estimation | multiple | R | NR | R | | NR | | AquaMap, GARP, GLM, GAM, MaxEnt | | | R | | | single | | | AUC, cross-validation ROC, Spearmann's rank | | | compared to survey data |  | | | | |
| **Reference** | **Application** | **Single/multiple** | **Occurrence data (n)** | **Bias in the occurrence data** | **Test/Train** | **Geographic extent** | | **Modeling algorithm** | | **Probability distribution** | | | **Thresholds** | | | **Precision test** | | | **Accuracy test** | | | |  | | | |
| Reside et al. 2010 | geographic range estimation | multiple | NR | RS | NR | | NR* | | MaxEnt | | | NR | | | NA | | | AUC; Wilcoxon signed rank tests | | | NR |  | | | | |
| Richmond et al. 2010 | methods, improve SDMs | multiple | R | RS | R | | Tailored | | MaxEnt | | | NR | | | multiple | | | AUC | | | ID |  | | | | |
| Rodda et al. 2011 | exotic species invasion | single | R | NR | R | | Tailored | | MaxEnt | | | R | | | NA | | | AUC, r2 w/AIC | | | Overlap |  | | | | |
| Rose and Wall 2011 | spatial epidemiology | single | R | NR | R | | NR* | | MaxEnt | | | R | | | single | | | AUC | | | NR |  | | | | |
| Sehgal et al. 2011 | spatial epidemiology | multiple | R | NR | NR | | tailored to a predetermined area | | MaxEnt, TREE, RF | | | NR | | | single | | | r | | | predictions compared to ID |  | | | | |
| Soberon 2010 | methods, illustrate a model | single | R | NR | NR | | tailored to a predetermined area | | Bioclim | | | NA | | | NR | | | NR | | | NR |  | | | | |
| Soria-Auza et al 2010 | methods,climatic variables | multiple | R | NR | R | | NER | | MaxEnt | | | NR | | | single | | | AUC | | | NR |  | | | | |
| Stiels et al. 2011 | exotic species invasion | single | R | RS | R | | Tailored by species | | MaxEnt | | | NR | | | multiple | | | AUC | | | NR |  | | | | |
| Taubmann et al. 2010 | conservation, climate change | single | R | NR | NR | | NER | | MaxEnt | | | NR | | | NR | | | AUC | | | 10-fold cross validation |  | | | | |
| Tognelli et al. 2010 | conservation, reserves | multiple | R | NR | R | | NER | | MaxEnt | | | NR | | | NR | | | AUC | | | NR |  | | | | |
| Tuanmu et al. 2011 | methods, incorporate remote sensing | single | R | NR | R | | tailored to a predetermined area | | MaxEnt | | | NR | | | NR* | | | AUC and MPA (minimum predicted area) | | | NR |  | | | | |
| Urbina-Cardona and Flores-Villela 2011 | conservation, reserves | multiple | R | NR | R | | NER | | MaxEnt | | | NR | | | NR | | | AUC | | | expert opinions' |  | | | | |
| Vaclavik et al. 2010 | spatial epidemiology | multiple | R | NR | NR | | tailored to a predetermined area | | MaxEnt | | | NR | | | single | | | AUC | | | ID |  | | | | |
| Vega et al. 2010 | spatial evolution | single | R | RS | NR | | tailored to a predetermined area | | MaxEnt, GARP | | | NR | | | single | | | AUC | | | Prange |  | | | | |
| Wilting et al. 2010 | conservation, species | single | R | NR | R | | Tailored by species | | MaxEnt | | | R | | | classes | | | AUC | | | Other algorithm |  | | | | |
